# Teaching Aquitard Concepts With Field‐Based High‐Resolution Head Profile Learning Activities

**DOI:** 10.1111/gwat.70042

**Published:** 2025-12-15

**Authors:** Jessica R. Meyer, Stephanie Tassier‐Surine, Bradley Cramer

**Affiliations:** ^1^ School of Earth, Environment, and Sustainability University of Iowa Iowa City Iowa; ^2^ Iowa Geological Survey Iowa City Iowa

## Abstract

Aquitards play critical roles in a variety of hydrogeologic processes. Despite their importance, coverage of aquitards in introductory hydrogeology textbooks is generally limited. This paper provides examples of classroom and field activities designed with an aquitard focus for instructors wishing to supplement textbook content. These activities emphasize high‐resolution head profiles. Examining head profiles prompts students to think about how aquitards influence head with depth and conversely how these plots can be used to delineate and characterize aquitards. During a classroom activity, students explore the connection between changes in vertical gradient and changes in hydraulic conductivity by sketching conceptual head profiles based on given boundary conditions and several aquifer/aquitard scenarios. In a companion field exercise, students measure high‐resolution head profiles using CMT multilevel systems at an outdoor learning laboratory. Students compare the high‐resolution head profiles to lower resolution profiles they obtain from clusters of conventional wells. The field exercise provides students with a tactile experience that can help build intuition for vertical head changes, practice interpreting aquitards from head profiles, and an example of how lower resolution head profiles may create uncertainty in aquitard delineation and vertical gradient estimates. A paleosol at the site forms a prominent aquitard providing a unique basis for discussions about the geology of aquitards and characteristics influencing aquitard integrity. Regardless of the approach used, incorporating more aquitard content into hydrogeology courses at all levels will be beneficial for future hydrogeologists tackling a range of issues from sustainable water supplies to waste disposal.

## Introduction

Aquitards, and more generally lower permeability materials, are critical components in many hydrogeologic processes operating at a variety of scales. At the flow system scale, aquitards substantially modify the trajectory of subsurface flow paths, increase groundwater residence times, and can have a strong influence on groundwater geochemistry within a flow system (e.g., Freeze and Witherspoon, 1967; Winter et al., 1998; Wang et al., 2013). Aquitards control the rates of flow between aquifers (e.g., Gardner et al., 2012) and can also serve as an additional source of water for confined aquifers being pumped (e.g., Bredehoeft et al., 1983). The properties and distribution of lower permeability materials are key to understanding and predicting subsidence (Galloway and Burbey, 2011; Bagheri‐Gavkosh et al., 2021), an issue with overwhelming social and economic costs (Kok and Costa, 2021). Aquitards, and low permeability media more generally, also play important roles with respect to the transport of contaminants from surface sources. Aquitards impede the migration of contaminants into underlying aquifers via physical and/or biogeochemical mechanisms (Cherry et al., 2006) and the diffusive transfer of contaminant mass from higher permeability materials into aquitards or disseminated low permeability materials has strong impacts on long‐term plume behavior and the success of remediation (e.g., Chapman and Parker, 2005; Parker et al., 2008). Aquitards also play a key role in protecting fresh groundwater resources from the upward migration of hazardous wastes (i.e., petroleum production fluids, industrial waste water, carbon dioxide) deliberately disposed of in the deep subsurface, and argillaceous rocks are being considered as hosts for the sequestration of radioactive materials (NEA, 2022).

Given their significance to many hydrogeologic problems, it seems reasonable to expect aquitards to feature prominently in introductory textbooks. However, these textbooks are often aquifer‐centric. A simple review of the table of contents and indices of common hydrogeology textbooks for topics describing the properties of and groundwater flow in aquifers and aquitards (or confining units) shows a strong dominance of aquifer entries. Instruction based on these textbooks, without supplementation, naturally focuses on teaching students to conceptualize and quantify groundwater flow in aquifers. For example, students learn to apply Darcy's Law to estimate flow rates and groundwater velocities in aquifers. They practice utilizing head distributions to determine lateral gradients and flow directions for aquifers. They learn techniques for analyzing hydraulic testing data to quantify aquifer hydraulic conductivity (*K*). In contrast, there is far less aquitard‐specific content in introductory hydrogeology textbooks. Sections on subsidence and consolidation are often the only areas with an emphasis on the properties of and groundwater flow in aquitards. When aquitards are discussed as a part of other topics, it is often in service of describing groundwater flow in the aquifers they confine. For example, aquitards are described in sections presenting analytical equations for the response of confined aquifers to pumping. The focus is on whether the aquitards are providing water to the pumped aquifer via transmission from adjacent aquifers and/or from aquitard storage (i.e., how “leaky” is the aquitard). For the purposes of developing these analytical equations, aquitards are represented as laterally extensive, uniform in thickness, homogeneous, and isotropic. In other words, aquitard characteristics are highly simplified and content describing aquitard characterization techniques is limited or entirely absent (Cherry et al., 2006). Consequently, without supplemental content, students may come away with a narrow view of what geologic materials might function as aquitards and lacking tools to conceptualize, delineate, and characterize these units.

The focus in introductory hydrogeology courses on aquifers is warranted given their global importance to water resources, but these aquifers do not exist in isolation, and students would also benefit from more aquitard‐specific content. This raises the question: how can more aquitard‐relevant topics be incorporated into already packed introductory hydrogeology courses? One way is to expand how we teach students to think about spatial distributions of hydraulic head. Often, the focus in undergraduate hydrogeology courses is on contouring head in two‐dimensional map view to represent aquifer equipotential surfaces and quantify lateral hydraulic gradients. These concepts are obviously important, but they do not provide students with perspective on how heads vary vertically within flow systems. This vertical perspective is often provided by having students construct flow nets for vertical cross‐sections and/or by showing students vertical cross‐sections with model‐derived hydraulic head contours (e.g., Tóth, 1963; Freeze and Witherspoon, 1967; Winter, 1978). In extreme examples, the vertical distribution of head is depicted by three‐dimensional stacking of equipotential surfaces from distinct aquifers or with diagrams showing the water level elevations in wells completed at different depths (e.g., Taylor et al., 2003). These methods of visualizing and thinking about head with depth all have their advantages but are complicated to produce and skip a simpler but similarly powerful approach: head profiles.

A head profile is a plot of hydraulic head on the *x*‐axis and depth or elevation on the *y*‐axis. Head profiles make the direction (upward or downward) of the vertical component of the hydraulic gradient (i.e., vertical gradient) immediately apparent. The slope of the head profile reflects the magnitude of the vertical gradient. However, if the head measurements are sparse and/or are blended across long depth intervals, as is common due to typical well designs, the vertical gradients will likely be biased low, the exact depths where the head change occurs will be uncertain, and changes in the direction of the vertical gradient might be obscured. These issues are remedied by deliberately using instrumentation that facilitates collection of numerous depth‐discrete, minimally blended head measurements along a single profile, or what is referred to as a high‐resolution head profile.

There are multiple ways to collect high‐resolution head profiles, particularly in component bedrock holes, including burying transducers at different depths in a borehole (e.g., Smith et al., 2013) or sealing a string of transducers hung at multiple depths into a borehole with a FLUTe liner (e.g., Pehme et al., 2014). Straddler packer systems may also be used to collect heads from numerous depths within a bedrock borehole (Gellasch et al., 2013). Although, with this approach, most of the borehole remains unsealed during each measurement, creating a potential for cross‐connective flow to influence the heads (Meyer and Parker, 2014). Commercially available, engineered multilevel systems (MLSs) (e.g., Westbay system, Waterloo system, Water FLUTe, and CMT) provide additional ways to collect high‐resolution head profiles in a wide variety of geologic/hydrogeologic settings and for a range of borehole constructions (Cherry et al., 2015). Engineered MLSs divide a single borehole up into numerous depth‐discrete monitoring intervals that can be used to measure heads, collect groundwater samples, and, in some cases, perform hydraulic tests. The CMT MLS (Einarson and Cherry, 2002) is an example of a low‐cost engineered MLS that provides up to 7 depth‐discrete monitoring intervals, can be designed and customized on site, and can be installed using standard backfilling techniques (https://www.solinst.com/instruments/multilevel‐systems/403‐cmt‐multilevel‐systems/). Although CMT MLSs are often used for contaminated site investigations, their low cost and use of standard installation techniques make them relatively accessible to instructors installing wells for teaching purposes.

High‐resolution head profiles provide direct and valuable insight into vertical components of gradient and variability in K with depth, making them valuable to aquitard‐focused studies (Cherry et al., 2006; Hart et al., 2008; Cohen and Cherry, 2020). Under steady‐state conditions, changes in the slope of a high‐resolution head profile indicate changes in the vertical component of *K* (*K*_*v*_). Aquitards, or units with relatively low *K*_*v*_, are immediately apparent as discrete sections of the head profiles with smaller slopes than the adjacent intervals (i.e., large changes in head across relatively small depth intervals). Studies in clayey aquitards (e.g., Goodall and Quigley, 1977; Desaulniers and Cherry, 1989; Keller et al., 1989; O'Shaughnessy and Garga, 1994; Filippini et al., 2020) and sedimentary rocks (e.g., Eaton et al., 2007; Meyer et al., 2014; Meyer et al., 2016; Runkel et al., 2018; Meyer et al., 2023; Medici et al., 2024) have demonstrated the utility of head profiles for identifying and delineating the boundaries and thicknesses of aquitards, providing insight into the properties of the geologic materials impeding vertical flow, and when head profiles are collected from several different locations in the same study area, assessing the lateral continuity of aquitards. High‐resolution head profiles have also been used in a variety of other ways to provide hydrogeologic insight such as calibrating numerical models used to estimate bulk *K*_*v*_, assessing the connectivity and persistence of hydraulically active fractures in aquifer and aquitard units, and evaluating the influence of sub‐ and supernormal pressures on regional groundwater flow (e.g., Raven et al., 1992; Gellasch et al., 2013; Smith et al., 2016; Meyer et al., 2023; Young et al., 2023).

Although high‐resolution head profiles are useful for studying aquitards specifically, and groundwater flow systems more generally, they are not typically depicted in introductory hydrogeology textbooks (Hart et al., 2008). A relatively recent book focused on visualizing and conceptualizing hydraulic head and groundwater flow by Cohen and Cherry (2020) makes important progress by describing the concept of a head profile, including a handful of conceptual head profiles, and providing several example problems/questions appropriate for beginning hydrogeology students. The objective of this issue paper is to provide one example of how field‐based head‐profile focused learning activities can be designed to advance students' understanding of aquitards, vertical distributions of head and flow in groundwater systems, and the potential bias introduced by long and/or poorly positioned well screens. A teaching field site was deliberately developed to support the incorporation of experiential learning into these activities. An example classroom exercise focused on predicting the influence of aquitards on head profiles is also provided that works well as a prelude to the field activities or as a stand‐alone module.

### Teaching Site Description

**Figure 1:**
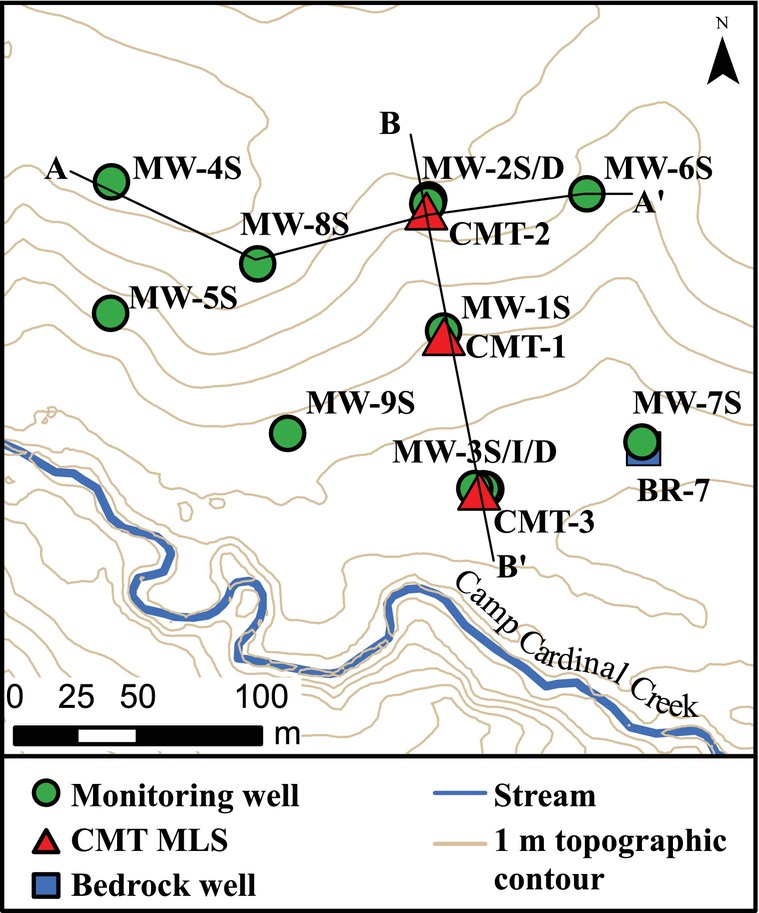
Map of APLL showing the topography, course of Camp Cardinal Creek, and locations of monitoring wells and CMT MLSs. Figures for cross‐sections A–A′ and B–B′ are provided in Data S1.

The Ashton Prairie Living Laboratory (APLL) is an outdoor teaching space about 4 km from the main University of Iowa campus and encompassing an area of roughly 8.5 acres. APLL is bounded on the south and west by Camp Cardinal Creek (Figure 1) and to the north by Clear Creek, both ultimately tributaries to the nearby (< 2 km) Iowa River. Topographically, the area slopes gently (~4%) from the northern boundary toward Camp Cardinal Creek (Figure 1). The proximity of Camp Cardinal Creek and the hillslope combine to create a valuable space for teaching a variety of concepts in soil science, geomorphology, hydrology, and hydrogeology.

Pleistocene deposits at APLL consist of several sequences of alluvial deposits, associated with the development of the Iowa River and Clear Creek, separated by periods of landscape stability and the formation of paleosols (Figure S1). The lower terrace consists of silt loam to sandy loam alluvial units with occasional coarse sand and gravel. A moderately well‐developed paleosol is formed in the terrace surface and consists of loam to silty clay loam with trace coarse sand and gravel. An overlying terrace sequence consists of undifferentiated loam and silty alluvial deposits. The associated paleosol is not as well‐developed and has a silt loam to loam texture with trace coarse sand and gravel. A later downcutting event on the southern part of the site eroded a portion of the upland and deposited a younger alluvial terrace at a lower elevation. The terrace is composed of at least 6 m of coarse sand and gravel at the base and is overlain by silty loam alluvium. No paleosol is present. The entire APLL site is mantled with 3 to 5 m of loess.

The APLL site hosts 1 existing bedrock well, 12 conventional monitoring wells, and 3 CMT MLSs. Boreholes for the conventional wells and CMT MLSs (Einarson and Cherry, 2002) were drilled using a hollow stem auger with the collection of continuous cores at each location. The cores were logged in detail, photographed, and sampled for laboratory grain size analysis and have been used to assess lithology and stratigraphy at APLL (Tassier‐Surine, 2025). The conventional wells include 1.5 or 3 m long screens either positioned across the water table in the loess or in the deeper alluvial materials (Table S1). Field logs of the cores and the water levels in the open boreholes were used to determine the depths and lengths for the monitoring well screens. These wells are purposefully distributed across the site to support learning exercises focused on basic hydraulic head measurements, development of water table maps, assessment of lateral groundwater flow directions, and slug testing. The CMT MLSs were installed to provide students with the opportunity to collect high‐resolution head profiles and use the results to delineate and describe potential aquitards.

The CMT MLSs are arranged from the top to the toe of the slope along a line oriented roughly parallel to a steady‐state lateral groundwater flow direction. Each CMT includes 7 depth‐discrete monitoring intervals between 0.15 and 1.28 m in length and specifically positioned based on the detailed core logs (Figure S1 and Table S1). The infrastructure at APLL has been deliberately developed to demonstrate the differences in understanding derived from a single MLS versus clustered wells completed at different depths by including conventional well clusters with the MLSs (Figure S1). Mowed paths are maintained between the wells to improve accessibility, and the most comprehensive cluster of wells and multilevel systems (Figure 1; MW‐3S/I/D and CMT‐3) was positioned at the toe of the slope just off the track to provide accessibility via a utility vehicle for people with mobility‐related issues.

## Teaching with Head Profiles

### Classroom Preparation

We have found it helpful to prepare students for the field activity by having them work through a conceptual exercise that introduces head profiles, vertical components of hydraulic gradient, and changes in vertical gradient in response to changes in *K*. Head profile exercises structured around theoretical permeameter experiments (Lambe and Whitman, 1969) and more generic aquifer/aquitard scenarios (Cohen and Cherry, 2020) were used as a starting point for the classroom activity described here. Students have already been introduced to hydraulic head, Darcy's Law, and the impact of changes in *K* on hydraulic gradient in previous lectures.

**Figure 2:**
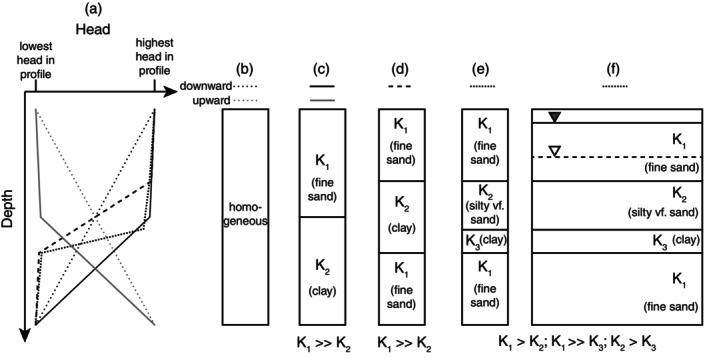
Examples of aquifer/aquitard layering and corresponding conceptual head profiles used for the classroom activity. Note that most of the head change occurs across the lowest *K* layer and that although the head profile looks vertical in the higher *K* units, there is a change in head/vertical gradient across these units.

The overall objective of the activity is for students to connect the changes in *K* with changes in vertical gradient across the profile; that is, to be able to explain how aquitards influence head profiles. To start, each group is provided with a worksheet (Conceptual Exercise in Data S2). The worksheet has a blank head profile with tick marks on the *x*‐axis representing the lowest and highest heads in the profile (Figure 2a). Directions on the worksheet instruct students to assume one‐dimensional vertical flow where the flow in must equal the flow out. The first question on the worksheet asks the students to draw a head profile representing an upward component of vertical gradient and another representing a downward component of vertical gradient for the case when the subsurface is homogeneous (Figure 2b). This first step is simple but helps students think about how head must vary with depth to create upward or downward vertical gradients prior to asking them to conceptualize the effect of a low *K* unit or aquitard on the head profile.

Then next step introduces aquitards into the exercise. The worksheet uses the same style of blank head profile with an added geologic column (or series of columns) alongside it. The degree of geologic complexity can vary from simple two (Figure 2c) or three (Figure 2d) layered systems through slightly more complicated representations of heterogeneity where there are several different contrasts in *K* rather than just one (Figure 2e). For each of these examples, students can be provided with information on the relative difference in *K* for each unit and/or they can be provided with textural/lithology terms that they then need to translate into relative *K* differences (Figure 2). If the topic of how secondary porosity features can increase bulk *K* has been covered, instructors can extend the exercise by indicating that a particular portion of a fine‐grained unit is intensely fractured while the remaining portion is unfractured. The worksheet includes a reminder of Darcy's Law and instructs students to sketch out a conceptual head profile for each of the geologic scenarios presented. With this approach, through collaboration and conversation (and some hinting and nudging from the instructor), students begin to conceptualize how the changes in *K* must influence the vertical component of the gradient, and thereby, effect the shape of the head profile.

It is also easy to incorporate other complementary groundwater flow concepts into this exercise. For example, if flow systems and the associated terminology of recharge (i.e., vertical component of flow is downward) and discharge (i.e., vertical component of flow is upward) areas have been covered, students can be instructed to draw a head profile representing a recharge or a discharge zone rather than providing explicit instructions to represent upward or downward vertical gradients. Similarly, if the concepts of unconfined and confined aquifers have been presented, geologic heterogeneity can be discussed using a cross‐sectional view with lines to represent the position of the water table and the confined unit's equipotential surface (Figure 2f). Students then have to use the relative elevation of the water table and potentiometric surface to determine if vertical gradients are upward or downward and then correctly represent the condition in the head profile.

The end of the exercise typically includes several follow‐up questions where students are asked to articulate, in writing, the concepts shown in the head profiles. Example follow‐up questions for an activity focused on the scenarios shown in Figure 2b and 2c include:

- How does head change as depth increases if the vertical component of the gradient is upward?
- Describe, using Darcy's Law, the difference between how you drew the trend in head with depth for the sand versus the clay.
- Describe how the head profile would change if the clay unit were replaced with a very fine‐grained sand unit?

This is a useful conceptual exercise that can be implemented at a variety of levels. However, the concept of head change with depth in a groundwater system and modification to the vertical gradient due to changes in K will still be relatively unintuitive to most students. Measurement of head profiles in the field can help students get over the intuition hurdle.

### Field Exercises

The following field exercises were co‐developed with the infrastructure installed at APLL but could be adapted for any teaching field site with multilevel monitoring and a vertical gradient. First, the class does a simple group activity where student volunteers demonstrate the decrease in head with depth at the CMT‐3 MLS by measuring water levels in the shallowest and deepest ports of the MLS (Field Exercise in Data S2). The activity is designed to help students build intuition for changes in head with depth. Next, the students are instructed to measure water levels in all the conventional wells at APLL and the single MLS assigned to their group. They use the conventional well heads to create a water table map in a separate exercise and this spreads the groups amongst the infrastructure so only one group occupies each MLS at a time. Before students split up and spread out, proper techniques for measuring water levels are demonstrated followed by students answering simple questions on their field lab worksheet (Field Exercise in Data S2) designed to get them thinking about the units of measure, resolution of their water level tapes, and sources of error and uncertainty in water level measurements.

**Figure 3:**
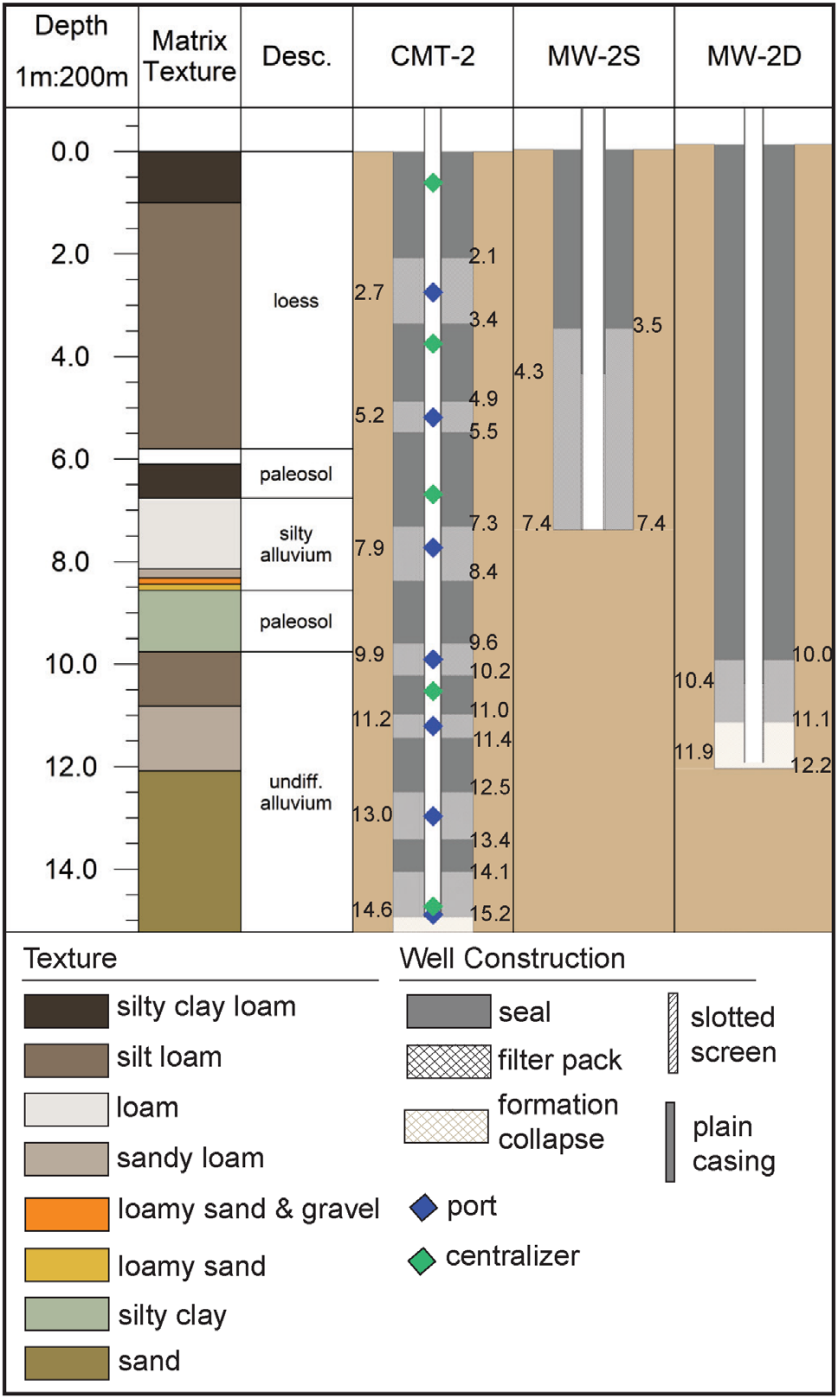
Example of the well construction data similar to what is provided to students for the field exercise.

We use an example from the CMT‐2 MLS to demonstrate the data students work with and questions they respond to for the remainder of the lab. The laboratory packet (Field Exercise in Data S2) includes the texture data derived from core logging and well construction diagrams and tables for the MLS and adjacent conventional wells (Figure 3). They need this information to plot the head profiles and answer the questions at the end of the lab and it also provides them with experience reading well construction diagrams.

**Figure 4:**
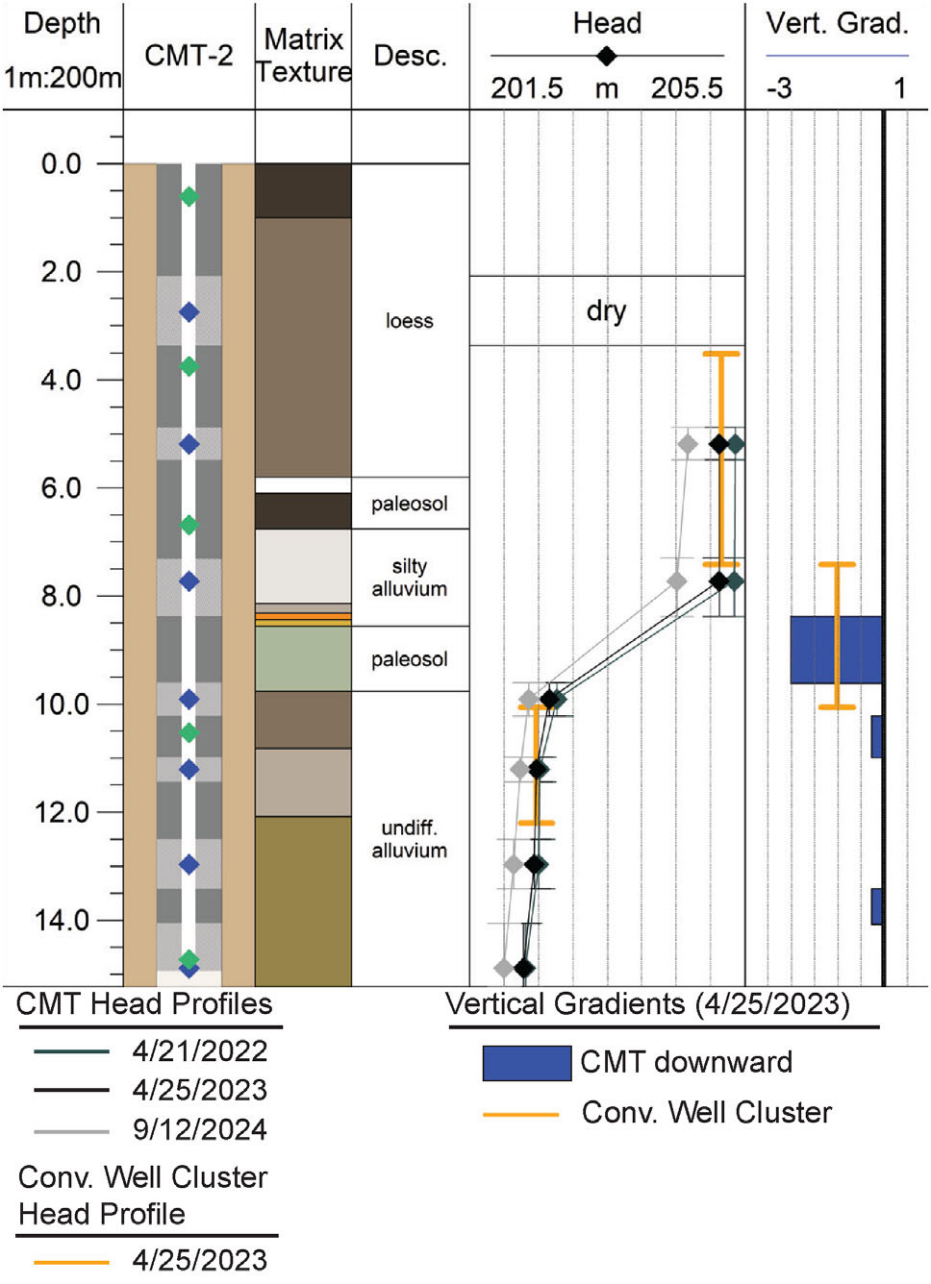
Example head profiles and vertical gradients from the CMT‐2 MLS and the associated cluster of conventional wells, MW‐2S and MW‐2D. A legend for the texture and well construction is provided in Figure 3.

First, students calculate heads for their MLS and associated conventional wells based on the provided reference elevations and the depths to water they measured. Next, they use the monitoring interval depths from the well construction figure and the heads they just calculated to plot the MLS head profile on graph paper. The CMT‐2 head profile shows a relatively large change in head with depth between 7.7 and 9.9 m below ground surface (bgs) and several smaller changes in head with depth deeper in the system (Figure 4). The version students are working with does not have the well construction, texture, or vertical gradient plotted adjacent to the head profiles. Those supporting items are provided here to help describe the activity. Once students plot the head profiles, they respond to a series of questions to guide them through the major observations. For example:

- According to the head profile you plotted for your MLS, does the head change gradually with depth or are the changes more abrupt?
- Calculate the vertical gradient for the largest change in head between two adjacent monitoring intervals (i.e., the most abrupt change).
- What might cause the most abrupt change in head observed in the profile?
- If you had to describe one depth interval in this profile as an aquitard which interval would it be (i.e., provide the top and bottom depths)? Explain how you know this interval impedes vertical flow compared with the adjacent intervals in the profile.

There are several key take aways from this part of the exercise. First, head changes are not gradual, which means vertical gradients and *K*_*v*_ are not uniform with depth. Second, segments with larger vertical gradients are impeding vertical flow and can likely be conceptualized as aquitards. In addition, the largest vertical gradient is greater than 1. This result can be unintuitive but is a natural result of subsurface heterogeneity (Hart et al., 2008). Lastly, it appears the paleosol between 8.6 and 9.8 m bgs coincides with and is likely responsible for the largest vertical gradient. This association creates a great opportunity to discuss the variability of aquitards and characteristics that contribute to aquitard integrity in a follow‐up classroom period. For example, we discuss the probability that the paleosol extends across the entire APLL and look at the core logs and core photos to determine if there is evidence for secondary porosity features (i.e., root holes, fractures, burrows, etc.) in the paleosol. We talk about how these features influence the hydrogeologic properties of the paleosol. The presence of another paleosol in the profile that does not seem to produce a head response is also fortuitous because it helps to highlight the importance of utilizing hydraulic data to delineate aquitards rather than relying on lithology or existing stratigraphy alone.

After the students have plotted the MLS head profile, their field lab packet prompts them to add the conventional well heads to the diagram. It is important to have them represent the conventional well data as a line segment or bar that extends across the entire monitoring interval (Figure 4). It will depend on how the wells are designed and the vertical gradient at the site, but often times comparison of the conventional well heads with the MLS heads in this way will make the blending of heads in the conventional well screen obvious. Additional questions, such as those listed below, target the impact of blending in longer open intervals on heads, vertical gradients, and delineation of aquitards.

- We discussed blending in lecture. Do you see evidence for blending based on the comparison of heads in the conventional monitoring wells and the multilevel systems? If so, describe where and how you know.
- How does the gradient you calculated for the sharpest change in head in the MLS compare to the gradient you calculated between the shallow and deep conventional well. If you did not have the data from the MLS and you were trying to estimate the velocity for the vertical component of flow, would your velocity be biased high or low? Why?
- Based on the conventional well head profile can you say with confidence which geologic feature is serving as an aquitard? Explain.
- What aspects of the MLS design AND construction help to avoid blending? Could those same principles also be applied to the conventional wells?

This part of the exercise shows students a real example of blending where the monitoring well data are biased, albeit slightly, toward the higher *K* sediments (Figure 4). This effect would be more extreme had the wells had longer screens, which are typical features of many wells, or if they had been positioned less deliberately. The exercise also demonstrates that low resolution data often result in underestimates of the vertical gradient, in this case by about 50% (Figure 4). Underestimated vertical gradients are common due to blending in long screens and overestimation of the thickness of the geologic feature producing the head change due to the low‐resolution nature of typical well clusters. Additionally, this exercise starts students thinking more broadly about what makes an aquitard and the importance of accurately delineating the depth across which the head change occurs to accurately quantify the gradient.

## Summary

This issue paper provides an example of a field‐based learning activity and complementary in‐class activity designed to incorporate more aquitard‐specific content into introductory hydrogeology courses. The approach involves expanding instruction on ways to visualize and interpret hydraulic head to include high‐resolution head profiles. When students measure, plot, and interpret high‐resolution head profiles, they connect directly with the influence aquitards have on the vertical component of gradient. These exercises guide students through how to interpret head profiles to identify the boundaries and thickness of aquitards within a sequence of geologic materials. The exercises also push the students to think about the variety of geologic materials that function as aquitards more broadly and facilitate discussion about aquitard integrity. The approach of using head profiles to teach students about aquitard concepts has additional benefits. For example, the students plot simple head profiles, which are a useful but uncommon way to visualize and interpret head distributions. The exercises also help students build intuition for changes in head with depth and components of vertical gradient and flow, concepts that are commonly confusing. Finally, students observe firsthand the uncertainty introduced into aquitard delineation and hydrogeologic data more generally by blending in long screens. If other instructors incorporate these activities into their courses, we would be happy to hear about any outcomes or improvements in student engagement and understanding that result. Regardless of the approach used, students will benefit from an educational foundation that includes aquitard‐specific learning objectives and activities because they play an important role in so many hydrogeologic problems.

## Supporting information


**Data S1.** Supporting Information.

**Data S2.** Supporting Information.

**Data S3.** Supporting Information.

## Data Availability

The data that support the findings of this study are available from the corresponding author upon reasonable request.
