# Supplementary material for: Teaching Aquitard Concepts With Field‐Based High‐Resolution Head Profile Learning Activities
Source: Ground Water. 2025 Dec 15;64(1):21–9. doi: 10.1111/gwat.70042 (PMC12857529; doi:10.1111/gwat.70042)
Supplement: Supplementary file 1 — Data S1. Supporting Information. [file GWAT-64-21-s001.pdf]

SUPPLEMENTARY INFORMATION

Figures, Tables, and Additional Details for Field Exercise

Teaching aquitard concepts with field-based high-resolution head profile learning activities

Jessica R. Meyer[1*], Stephanie Tassier-Surine[2], Bradley Cramer[1]

[1] School of Earth, Environment, and Sustainability, University of Iowa, Iowa City, IA, 52242

[2] Iowa Geological Survey, Iowa City, IA

* corresponding author

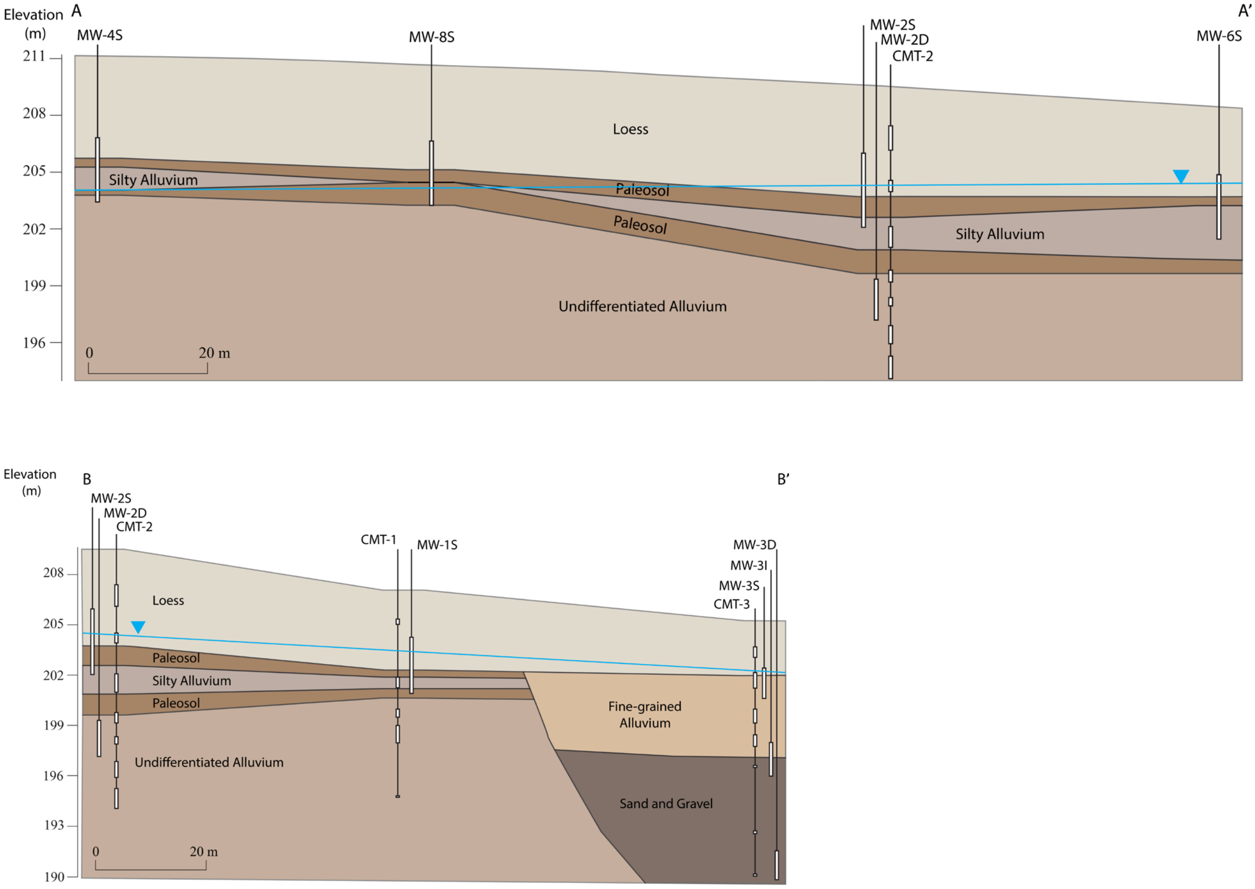

Fig. S1 – Conceptual east-west (upper panel) and north-south (lower panel) cross-sections showing the primary geologic units at APLL, the position of the conventional well and multilevel system monitoring intervals, and the water table elevation in relatively wet years. Cross-sections were modified from Tassier-Surine (2025) Figures 19 and 20. Lines of section are noted in Fig.1 in the main manuscript.

Table S1 – Construction details for the 3 CMT MLSs and conventional monitoring wells shown in cross-section in Fig. S1.

Station ID[1]	Interval ID	Port Depth m bgs	Monitoring Interval[2]		Comment
			Top Depth m bgs	Bottom Depth m bgs	
CMT-1	1	1.98	1.68	1.98	
	2	3.66			Bentonite bridged and sealed the port
	3	5.49	5.11	5.72	
	4	7.32	7.01	7.47	
	5	8.53	7.92	8.99	
	6	9.98			Bentonite bridged and sealed the port
	7	12.19	12.14	12.19	
MW-1S	0	n/a	2.74	6.10	
CMT-2	1	2.71	2.07	3.35	
	6	5.18	4.88	5.49	
	5	7.86	7.31	8.38	
	4	9.91	9.60	10.21	
	3	11.22	10.97	11.43	
	2	12.95	12.50	13.41	
	7	14.63	14.05	15.24	
MW-2S	0	n/a	3.51	7.44	
MW-2D	0	n/a	10.06	12.19	
CMT-3	1	1.86	1.52	2.13	
	2	3.38	3.05	3.96	
	3	5.46	5.18	6.02	
	4	6.89	6.71	7.39	
	5	8.56	8.49	8.64	
	6	12.45	12.37	12.53	
	7	14.97	14.92	14.97	
MW-3S	0	n/a	2.74	4.57	
MW-3I	0	n/a	7.16	9.14	
MW-3D	0	n/a	13.50	15.24	
MW-4S	0	n/a	4.27	7.62	
MW-6S	0	n/a	4.18	7.53	
MW-8S	0	n/a	4.27	7.62	

[1] All conventional monitoring wells ('MW') are constructed from 2-inch schedule 40 PVC with 0.01 inch slotted screened excepting MW-2D. MW-2D is constructed from 1.5-inch schedule 40 PVC with a 0.01 inch slotted screen. All CMT MLSs are 7 port systems with an outer diameter of 1.7-inches.

[2] Monitoring intervals shaded grey represent the top and bottom depths of the screen in instances where the formation materials collapsed around the well. All other monitoring intervals represent the top and bottom depths of the sand packs.

Field Exercise Group Activity - Building Intuition for Changes in Head with Depth

The first part of the field exercise is a group activity focused on helping students build intuition for changes in head with depth. The entire class gathers around an MLS, in this case, CMT-3 because its most accessible (Fig. 1 – main manuscript). A student volunteer is then asked to measure the depth to water in the tube corresponding to the shallowest monitoring interval in the system (i.e., interval 1). By design, the shallowest port at APLL is often dry. So, they move to monitoring interval 2 which is typically just below the water table. This is a good time to ask the group what hydrogeologic feature occurs between the two monitoring intervals. It is important for them to understand the water level elevation in monitoring interval 2 most closely corresponds to the water table. Next, the volunteer student is asked to carefully measure the water level in monitoring interval 2 again. This time, when the tape beeps they are instructed to pinch the tape at the reference point and not let go. Then, they are instructed to pull the tape out (all while continuing to pinch the tape where it indicated the depth to water) and slowly start moving it down the tube corresponding to monitoring interval 7, the deepest zone in the MLS. If the vertical component of gradient is upward, the class will hear the beep before the student's hand reaches the reference point and if its downward their hand will advance all the way to the reference point and the tape will not beep. Many students are genuinely surprised by this result and curious if something is wrong with the measurement. This moment of confusion provides an opportunity to discuss the outcome. Why isn't the water level elevation at depth equal to the elevation of the water table? What does this tell us about the potential for vertical flow at this location? Then, other members of the class are encouraged to give it a try.

References

Tassier-Surine, S. 2025. Advances in Quaternary Stratigraphy, Unit Correlation, and Event Timing in Iowa. PhD Thesis, Department of Earth and Enviromental Science, University of Iowa, Iowa City, Iowa.
