# Supplementary material for: Teaching Aquitard Concepts With Field‐Based High‐Resolution Head Profile Learning Activities
Source: Ground Water. 2025 Dec 15;64(1):21–9. doi: 10.1111/gwat.70042 (PMC12857529; doi:10.1111/gwat.70042)
Supplement: Supplementary file 2 — Data S2. Supporting Information. [file GWAT-64-21-s002.pdf]

*SUPPLEMENTARY INFORMATION*

*Example Worksheet for Classroom Conceptual Exercise*

# Teaching aquitard concepts with field-based high-resolution head profile learning activities

Jessica R. Meyer[1*], Stephanie Tassier-Surine[2], Bradley Cramer[1]

[1] School of Earth, Environment, and Sustainability, University of Iowa, Iowa City, IA, 52242

[2] Iowa Geological Survey, Iowa City, IA

* corresponding author

Conceptual Exercise – Head Profiles

Name: ________________________ _______/ XX pts

_____5 pts 1. The figure below provides the axis for a plot of head (on the x-axis) versus depth (on the y-axis). Depth increases down the page along the y-axis and head increases to the right on the x-axis. Two tics on the x-axis show the values for the highest and lowest heads in the profile. Alongside the head profile plot is a column describing the subsurface geology corresponding to the depths on the y-axis. **Assume flow through the system is entirely vertical and flow in equals flow out.**

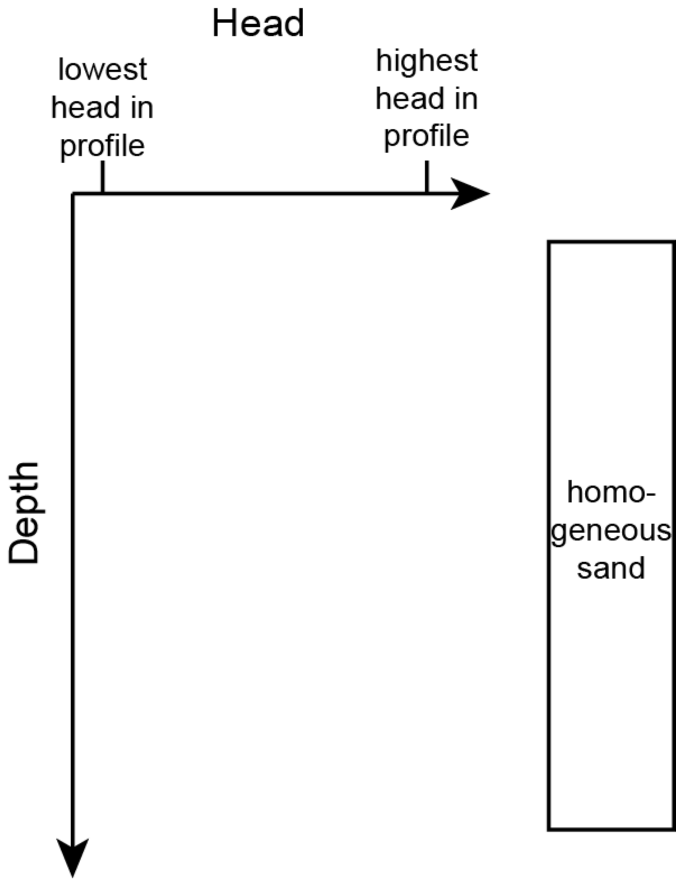


a) Draw a *solid* line on the head profile plot to show how head changes versus depth if the hydraulic gradient is upward. *(X pts)*

b) Draw a *dashed* line on the head profile plot to show how head changes versus depth if the hydraulic gradient is downward. *(X pts)*

c) Explain, in your own words, how the head change with depth for the upward gradient scenario is different from the head change with depth for the downward gradient scenario. *(X pts)*

_____6 pts 2. The figure below provides an empty plot for a head profile and a column describing the subsurface geology corresponding to the depths on the y-axis. **Assume flow through the system is entirely vertical and flow in equals flow out.**

**REMEMBER:** $\boldsymbol{q = -K\frac{\Delta h}{\Delta l}}$

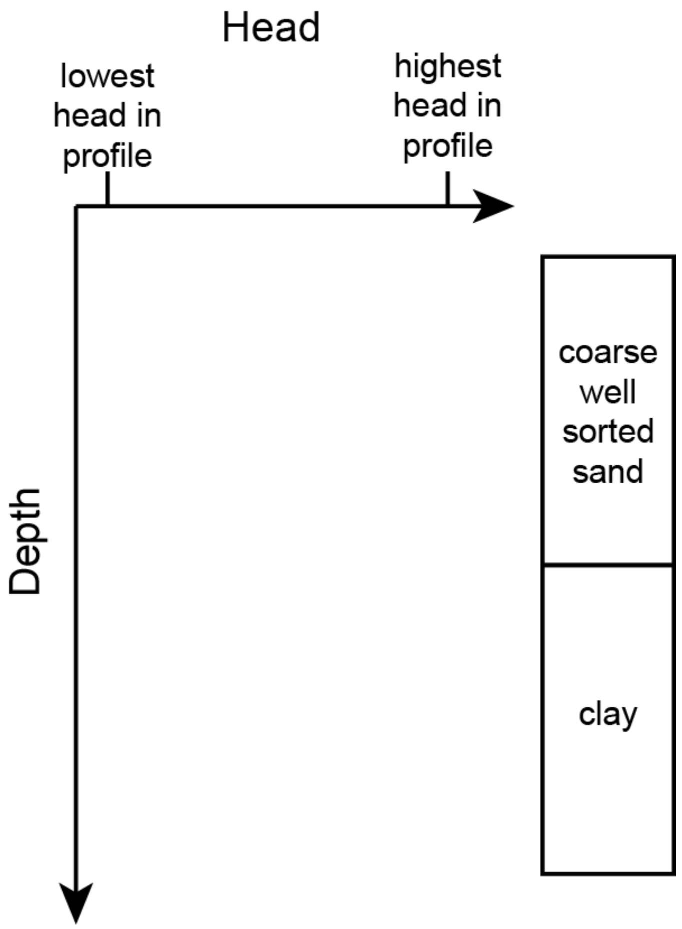


*a)* What hydrogeological parameter would be orders of magnitude different between the coarse, well sorted sand and the clay? *(X pts)*

b) Assume the hydraulic gradient is upward. Sketch in a line showing **conceptually** how the head would change versus depth through the sand and how the head would change versus depth through the clay. *Note*: the head at the very bottom of the sand must be equal to the head at the very top of the clay. (*X pts*)

c) Describe, using Darcy's Law, the difference between how you drew the line for the sand and how you drew the line for the clay. (*X pts*)
