# Supplementary material for: Teaching Aquitard Concepts With Field‐Based High‐Resolution Head Profile Learning Activities
Source: Ground Water. 2025 Dec 15;64(1):21–9. doi: 10.1111/gwat.70042 (PMC12857529; doi:10.1111/gwat.70042)
Supplement: Supplementary file 3 — Data S3. Supporting Information. [file GWAT-64-21-s003.pdf]

SUPPLEMENTARY INFORMATION

Example Lab Packet for Field Exercise

Teaching aquitard concepts with field-based high-resolution head profile learning activities

Jessica R. Meyer[1*], Stephanie Tassier-Surine[2], Bradley Cramer[1]

[1] School of Earth, Environment, and Sustainability, University of Iowa, Iowa City, IA, 52242

[2] Iowa Geological Survey, Iowa City, IA

* corresponding author

Lab: Collecting Water Levels, Measuring Changes in Head with Depth, and Delineating Potential Aquitards

Learning Objectives:

- Be able to use water level tapes to collect accurate water levels from conventional wells and CMT multilevel systems.
- Describe common sources of error in water level measurements.
- Practice calculating total hydraulic head from water level measurements and well construction information.
- Practice plotting hydraulic head versus depth.
- Use a head profile to determine the direction of the vertical gradient (upward or downward) from the plot and identify potential aquitards.
- Describe the concept of 'blending' in wells with long open intervals.

Background

Today you will be collecting water levels (also called depth to water measurements) from all the conventional monitoring wells and from 1 CMT multilevel system at the Ashton Prairie Living Laboratory (APLL). We will use these water levels for this and several subsequent exercises.

For this exercise, you will calculate the hydraulic head for each interval in the CMT multilevel system that you measured and for the 2-3 conventional wells co-located with the CMT. Then you will prepare a plot of hydraulic head versus depth using these data. You will use your observations during water level data collection, the heads you calculated, and your plot to answer the questions at the end of the lab.

Materials

Each group will be provided with a standard water level tape to measure water levels in the conventional wells. Each CMT multilevel system will have a coaxial (small diameter) water level tape stationed next to it for measuring water levels in those systems.

In addition to this lab handout, you will be provided with a package of information for the wells and multilevel systems at APLL. This package of information includes:

- a table that lists all the wells and multilevel at the site, the water level measurement reference point at each well/multilevel system, and the elevation of the water level reference points
- a map showing the location of each of the conventional wells and multilevel systems at APLL
- well construction information for the CMT multilevel systems and the conventional wells co-located with them
- profiles of basic lithology versus depth for each CMT multilevel system location

Instructions

1. Group discussion and short activity.
2. Answer questions 1-2 of the lab.
3. Split up into groups of two or three.
4. Measure water levels in the wells/multilevel systems listed below. Make sure everyone in your group gets multiple opportunities to make the measurements and record the data.
    a. All the conventional monitoring wells (12 total)
    b. The open bedrock well

c. All seven of the monitoring intervals in the CMT MLS assigned to your group

5. As you measure the water levels, record the data in the table included with the lab.
   a. Record your measured water levels in the table.
   b. Remember to fill out the date, personnel, time, unit, and reference information.
   c. You will calculate the heads and fill in that column in the table when you answer the questions at the end of the lab.
6. Once you have measured all your water levels, return to the CMT-3 location.
7. Answer questions 3-12 of the lab.

Name:__________________________________ ________ pts out of XX

Questions

1. What is the unit of measure for your water level tape? What is the magnitude of the major and minor graduations on your water level tape *(XX pts)*

**Unit of measure**	
**Major graduations**	
**Minor graduations**	

2. Can you use your water level tape to distinguish the difference between a water level of 25.568 and 25.569? Explain why or why not. *(XX pts)*

3. Based on your experience at the APLL today, describe two potential sources of error in your water level measurements. *(XX pts)*

4. Calculate the water level elevations (i.e., total heads) in ft above mean sea level (AMSL) for the CMT you measured and the conventional wells clustered with it. Record the heads on your data sheet. *(XX pts)*

5. Using the graph paper provided, plot the head for monitoring interval 1 in your CMT versus the midpoint depth of monitoring interval 1. Repeat the process for the other 6 monitoring intervals on the same graph paper. Join the points together with a line to better visualize the changes in head with depth. (*XX pts)*

6. Look at the head profile. Is the vertical gradient upward or downward? Explain how you know.*(XX pts)*

7. Calculate the vertical gradient (change in head/change in distance) for the largest change in head between two adjacent monitoring intervals in your head profile (i.e., the most abrupt change). (*XX pts*)

8. What is the texture of the geologic materials coincident with the depth interval from question 7 and how is the texture different from the intervals just above and below? *(XX pts*)

9. If you had to describe one depth interval in this profile as an aquitard which interval would it be (i.e., provide the top and bottom depths)? Briefly explain why you think this interval impedes vertical flow compared to the adjacent intervals in the profile. *(XX pts)*

10. On the same piece of graph paper, plot the head for the shallow conventional well co-located with your CMT (either MW-2S or MW-3S) as a line that extends from the top depth to the bottom depth of the monitoring interval. Do the same thing for the deep conventional well co-located with your CMT (MW-2D or MW-3D). Join the heads for the shallow and deep well with a line to make a head profile *(XX pts)*

11. Estimate the vertical gradient based on the data from the shallow and deep conventional wells. HINT: use the distance from the bottom of the shallow well's monitoring interval to the top of the deep wells monitoring interval in your calculation. Why is this estimate of the vertical gradient different from the one you calculated for question 7? *(XX pts)*

12. Based on the conventional well head profile can you say with confidence which geologic feature is serving as the primary aquitard? Briefly explain. (*XX pts*)

Map showing well and CMT multilevel system locations at APLL

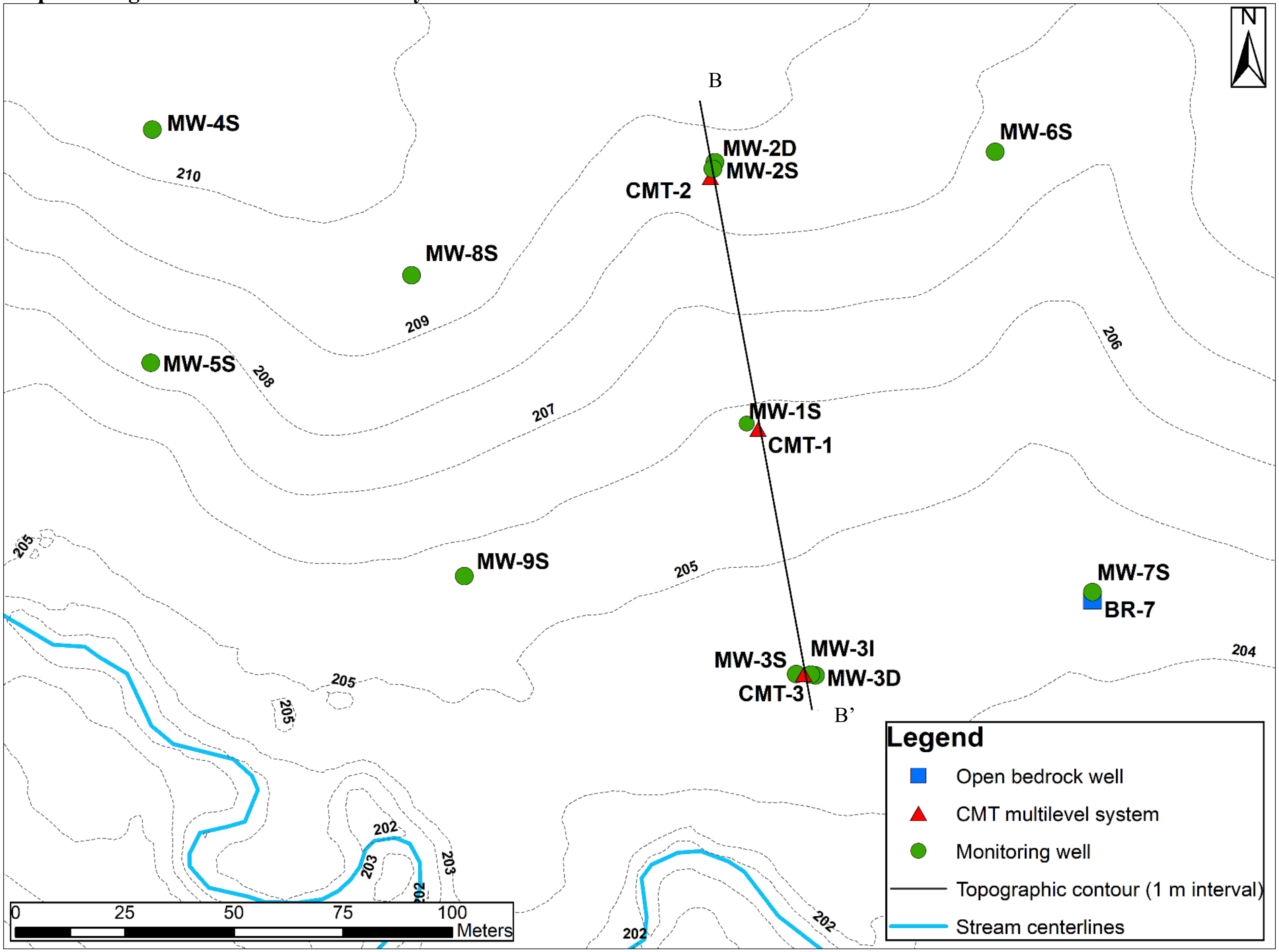

Date:____________________________

Personnel:________________________

Station ID	Interval	Time (24 hr)	Water Level	Water Level Unit	Water Level Reference	Reference Elev ft AMSL	Water Level Elevation (Head) ft AMSL	Comments
MW-1S	0							
MW-2S	0							
MW-2D	0							
MW-3S	0							
MW-3I	0							
MW-3D	0							
MW-4S	0							
MW-5S	0							
MW-6S	0							
MW-7S	0							
BR-7	0							
MW-8S	0							
MW-9S	0							
CMT-2	1							
CMT-2	2							
CMT-2	3							
CMT-2	4							
CMT-2	5							
CMT-2	6							
CMT-2	7							

Basic information for conventional wells and multilevel systems at APLL

Station ID	Station Type	Water Level Reference Point	Reference Point Elevation (ft AMSL)
MW-1S	Monitoring Well	Black Cap Coupling	682.40
MW-2S	Monitoring Well	Black Cap Coupling	690.35
MW-2D	Monitoring Well	1.5inCasing	690.22
MW-3S	Monitoring Well	Black Cap Coupling	676.14
MW-3I	Monitoring Well	Black Cap Coupling	676.05
MW-3D	Monitoring Well	Black Cap Coupling	676.68
MW-4S	Monitoring Well	Black Cap Coupling	695.7
MW-5S	Monitoring Well	Black Cap Coupling	686.35
MW-6S	Monitoring Well	Black Cap Coupling	687.09
MW-7S	Monitoring Well	Black Cap Coupling	676.07
BR-7	Open Bedrock Well	5inCasing	674.18
MW-8S	Monitoring Well	Black Cap Coupling	693.29
MW-9S	Monitoring Well	Black Cap Coupling	680.29
CMT-2	CMT MLS	Top of CMT Tube	690.38
CMT-3	CMT MLS	Top of CMT Tube	676.53

Well Construction Information for CMT-2, MW-2S, and MW-2D

Station ID	Interval	Top of Interval Depth (ft bgs)	Bottom of Interval Depth (ft bgs)	Midpoint of Interval Depth (ft bgs)
MW-2S	0	11.5	24.4	17.9
MW-2D	0	33.0	40.0	36.5
CMT-2	1	6.8	11.0	8.9
CMT-2	6	16.0	18.0	17.0
CMT-2	5	24.0	27.5	25.8
CMT-2	4	31.5	33.5	32.5
CMT-2	3	36.0	37.5	36.8
CMT-2	2	41.0	44.0	42.5
CMT-2	7	46.1	50.0	48.0

Texture (grain size) of geologic materials and Well Construction for MW-2S, MW-2D, and CMT-2

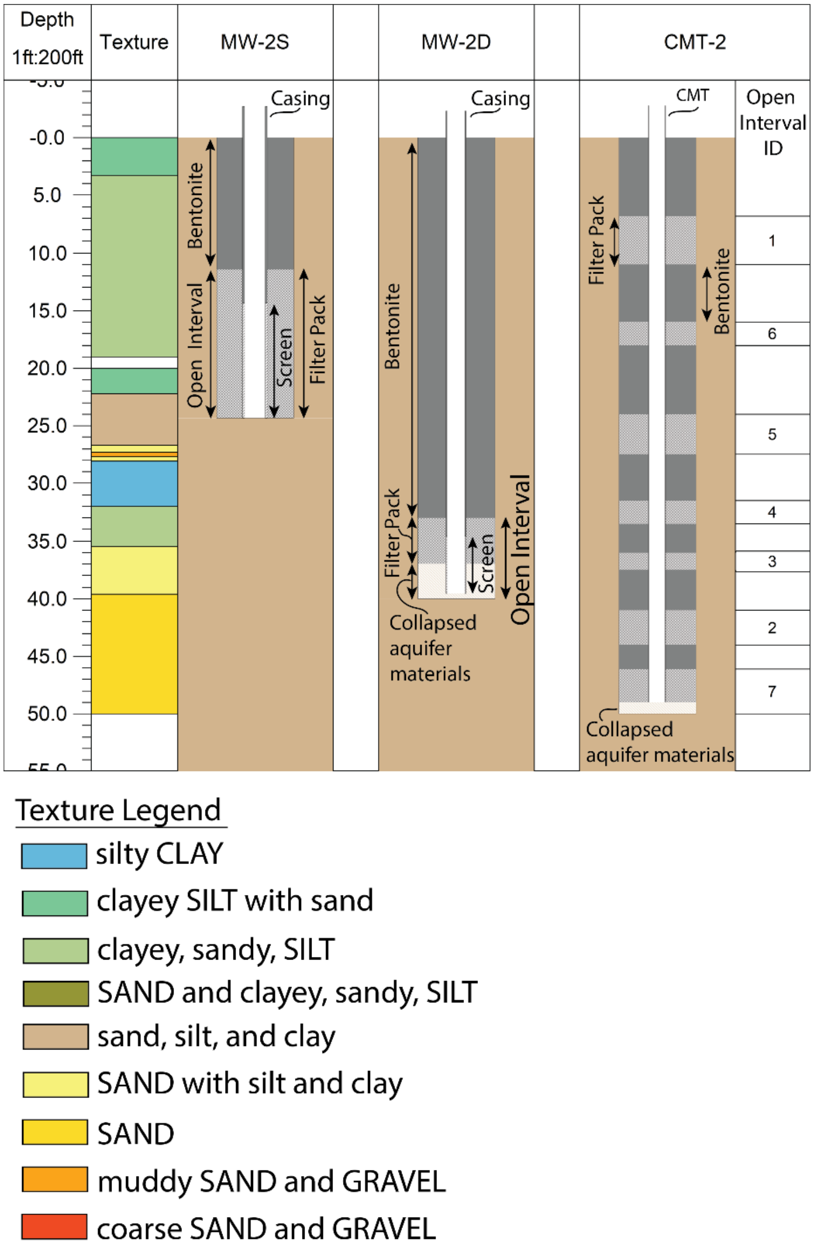

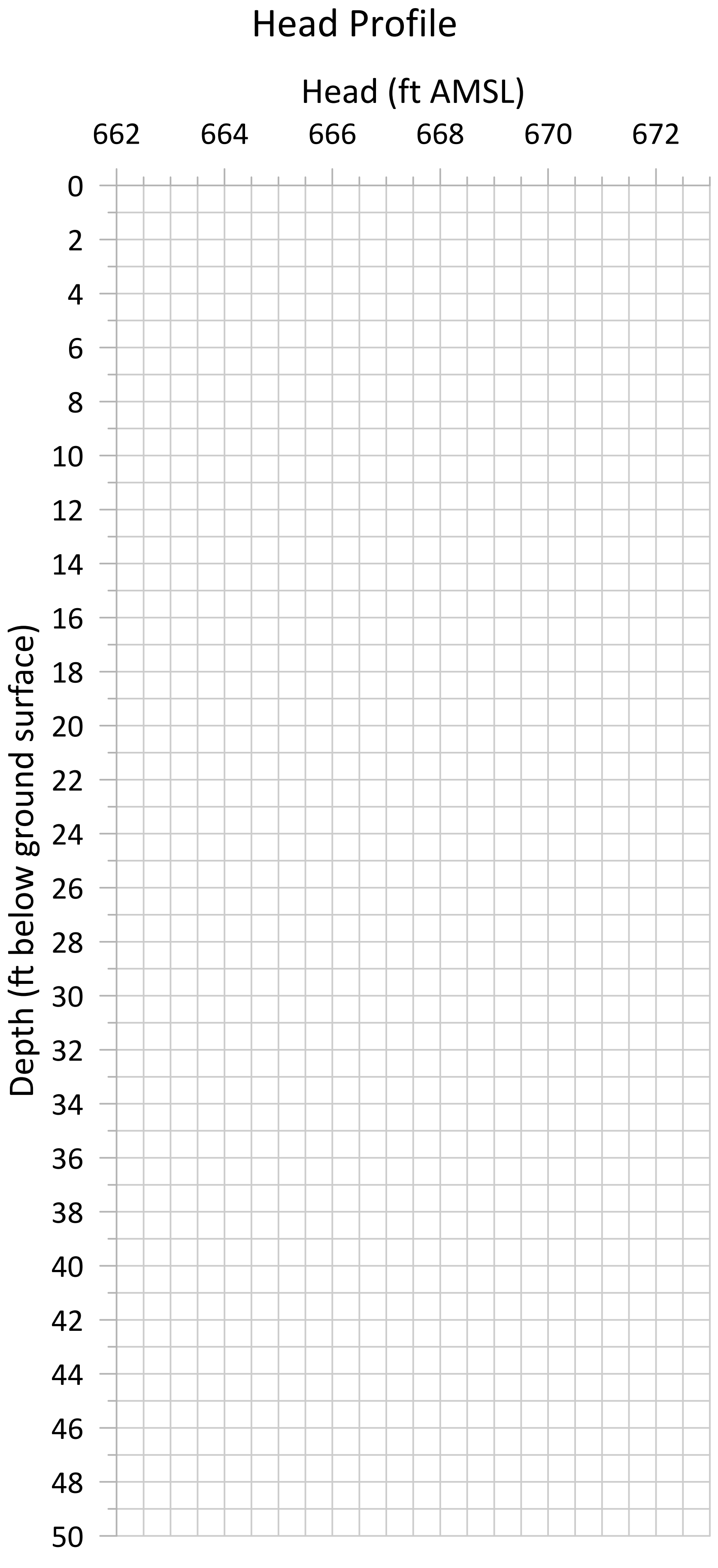
Head Profile
Head (ft AMSL)
662
664
666
668
670
672
Depth (ft below ground surface)
0
2
4
6
8
10
12
14
16
18
20
22
24
26
28
30
32
34
36
38
40
42
44
46
48
50
